# NEXAFS study of electronic and atomic structure of active layer in Al/indium tin oxide/TiO_2_ stack during resistive switching

**DOI:** 10.1080/14686996.2016.1182851

**Published:** 2016-06-24

**Authors:** Elena Filatova, Aleksei Konashuk, Yuri Petrov, Evgeny Ubyivovk, Andrey Sokolov, Andrei Selivanov, Victor Drozd

**Affiliations:** ^a^ Institute of Physics, St Petersburg State University, Ul’yanovskaya Str. 1, Peterhof, 198504, St Petersburg, Russia; ^b^ Institute Nanometre Optics and Technology (FG-INT), Helmholtz-Zentrum Berlin für Materialien und Energie GmbH, Albert Einstein Str. 15, 12489, Berlin, Germany

**Keywords:** ReRAM, NEXAFS, current instability, local heating, molecular oxygen

## Abstract

We have studied the stability of the resistive switching process in the Al/(In_2_O_3_)_0.9_(SnO_2_)_0.1_/TiO_2_ assembly grown by atomic layer deposition. Besides electrical characterization the effect of electric field on the atomic electronic structure of the TiO_2_ layer was studied using near edge X-ray absorption fine structure (NEXAFS) spectroscopy. The region of the current instability in the I-V characteristics was revealed. Presumably this current instability is supported by the amorphous structure of the TiO_2_ film but is initiated by the surface morphology of the Al substrate. A formation of the O_2_ molecules was established which occurs specifically in the region of the current instability that is a result of electrical Joule heating manifestation.

## Graphical abstract

**Figure:**
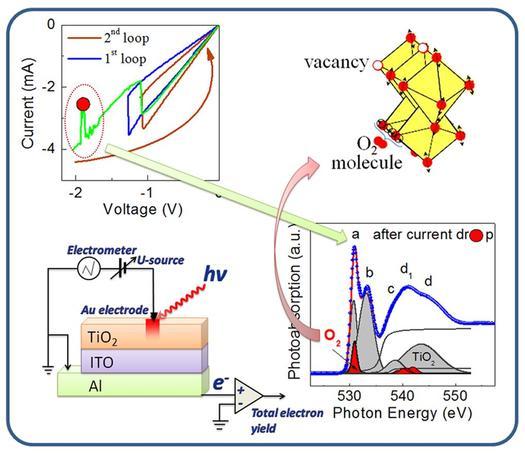

Electrophysical and NEXAFS studies were implemented in the same point of Al/ITO/TiO_2_ assembly (left bottom corner). The region of the current instability in the I-V characteristics was revealed. The current instability resulted in transition to a new hysteresis loop (left upper corner). A formation of the O_2_ molecules in the volume of TiO_2_ active layer has been established specifically in the region of current instability (right upper and bottom corners).

## 1. Introduction

Resistance-change random access memory (ReRAM) has attracted extensive attention as a promising candidate for non-volatile memory due to its high switching speed, high scalability, multi-bit storage potential and simple structure.[1–14] The principle of operation of ReRAMs involves a functional dependence of resistance of the material on the charge passing through it [15] that is realized in devices as resistive switching.[16] There is consensus now that resistive switching is performed by the conductive filaments formation/rupture process in many systems, and the conductive filament model is now accepted extensively.[3,4,17–21] Note that conductive filaments need to be initially formed in the insulator.[22,23] However, there still exist some issues in ReRAM devices based on the filament formation/rupture process that are critical to guarantee the stability and reproducibility of the operations in the ReRAM. Currently, [16,24–26]the possible causes for the lack of stability of resistance values in the low resistive state (R_L_) and high resistive state (R_H_) are being actively discussed.[16,24–26] It is noted [24,25,27] that the low resistance state is more stable than the high resistance state. According to the model proposed in [28], the distribution of filaments in the switching matrix is generally random, allowing a large variation of switching voltages for different cells from time to time. One of the factors affecting the stability of the resistive switching is a microstructure of the active layer.[29–31] In these references it was found that with increase of crystallinity (decrease of grain boundary density), the resistances and switching voltages become much more stable. According to [32], the location and growth direction of the filaments are confined by the grain boundaries.

The goal of the current paper is a study of the stability of the resistive switching process in the Al/ITO/TiO_2_ (ITO is (In_2_O_3_)_0.9_(SnO_2_)_0.1_) assembly. Earlier [33] we revealed that metal/TCO/TiO_2_ (TCO is a transparent conducting oxide) assemblies demonstrate the memristor effect after synthesis (without additional annealing) that provides progress towards understanding the nature of the memristor effect. Also it was established that after the switching process these structures can be retained for a long time. The production-grade Al wafer has been chosen as the substrate because of the specific morphology of its surface (grainy morphology). This allows us to analyze the effect of the surface morphology on the microstructure of the film and as a consequence on the stability of the resistive switching in the system. As a reference system the deg-Si/ITO/TiO_2_ assembly was used, where deg-Si is degenerate silicon substrate with atomically smooth surface and electrical properties nearly the same as those of a metal substrate. In order to gain insight into the electronic and atomic structure of the TiO_2_ film in addition to electrophysical investigations we have carried out near edge X-ray absorption fine structure (NEXAFS) spectroscopic studies of the assemblies after applying the electric field. We have studied the effect of the electric field at different stages, characterized by different values of current and voltage, on the atomic and electronic structure of assemblies. The NEXAFS arises from the excitations into unoccupied molecular orbitals. NEXAFS is dominated by multiple scattering of a low-energy photoelectron in the valence potential set up by the nearest surroundings. Spectral “fingerprint” techniques can be used to identify the local bonding environment. This fact defines the highest sensitivity of NEXAFS, to distinguish chemical bonds and nearest neighbors. Thus NEXAFS provides information about local (associated with a hole localization in the core shell) and partial (allowing for certain angular momentum symmetry) electronic density of states of the conduction band.

## 2. Experimental methods

TiO_2_ film of 20 nm thickness was grown by the atomic layer deposition (ALD) technique on production-grade Al and deg-Si wafers. ALD provides thickness control and aggressive conformality of TiO_2_ thin films at a reasonably low processing temperature. The TiO_2_ film was deposited in a cross-flow “Nanoserf” reactor at a temperature of 200°C using TiCl_4_, Ti[OCH(CH_3_)_2_]_4_, [(CH_3_)_2_ N]_4_Ti and H_2_O precursors. A homogeneous ITO buffer layer of thickness 100 nm was grown on the Al and deg-Si substrates prior to the ALD deposition of TiO_2_ film.

The surface topography of Al/ITO/TiO_2_ and deg-Si/ITO/TiO_2_ assemblies was examined with the helium ion microscope (HIM) at a chamber base pressure of 10^−7^ Torr. A focused beam of single-charged helium ions accelerated to 35 keV scanned over selected area of the sample. A beam current from 0.1 to 1 pA was used for imaging to avoid ion-induced sputtering of the sample. Images were obtained in the secondary electron detection mode with a conventional Everhart-Thornley detector. The high resolution transmission electron microscopy (HRTEM) images were obtained with a Carl Zeiss Libra 200 FE 200 keV microscope.

**Figure 1.**
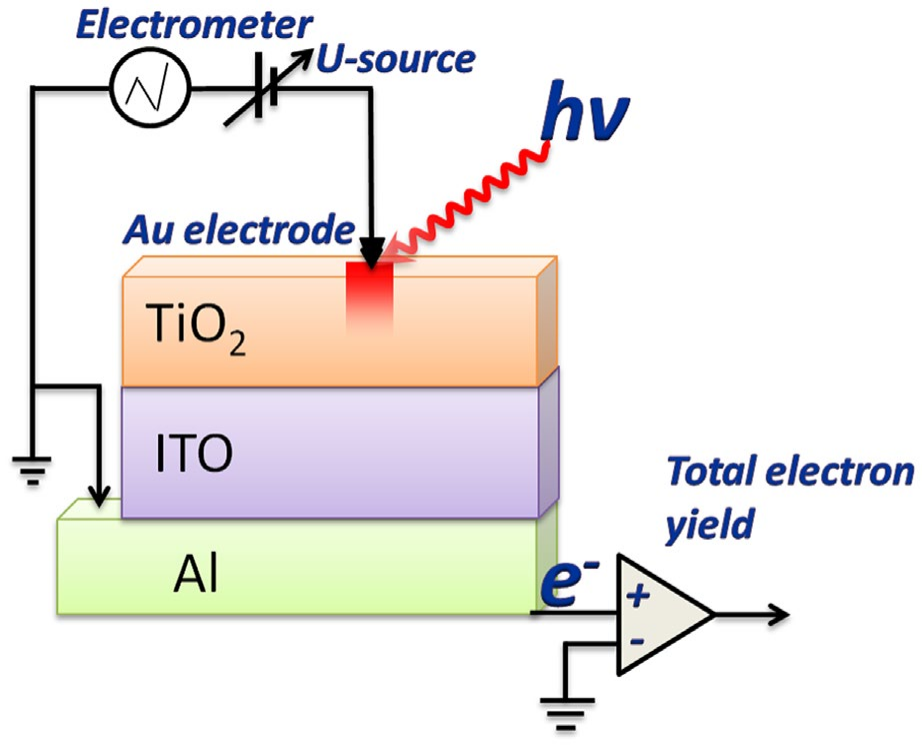
Schematic representation of electrophysical and NEXAFS measurements implemented at the same point of the sample.

**Figure 2.**
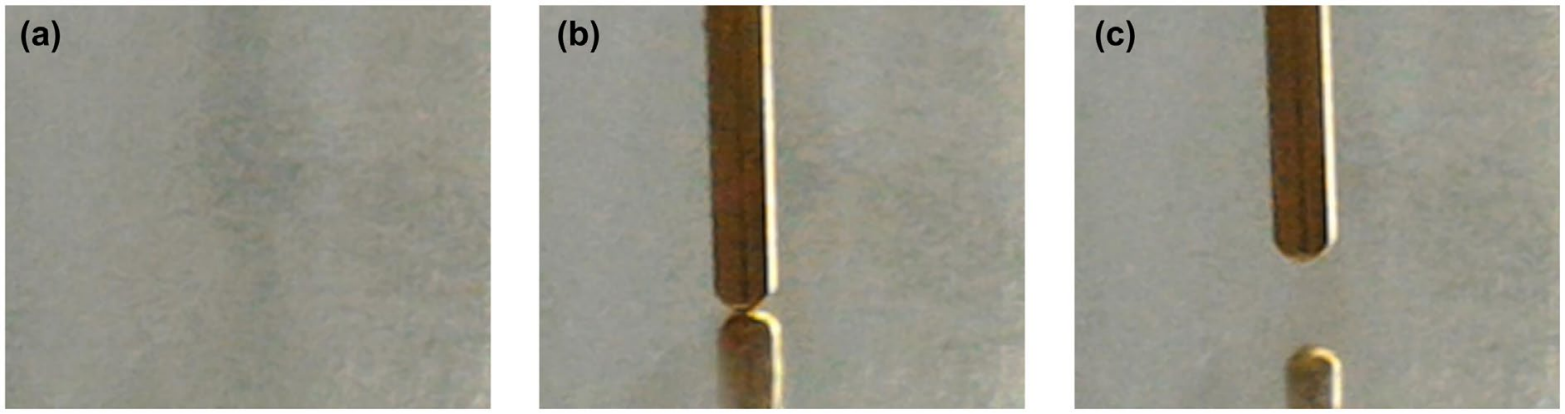
Photograph of the sample surface before and after electrode landing and retraction: sample placed on measuring table (a), electrode landed (b), electrode retracted (c). The diameter of electrode was about 1 mm, the contact area was about 1 × 10^−4^ cm^2^.

The I-V characteristics of the synthesized structures were measured at room temperature at ambient atmosphere using an original setup in the I-V sweep mode. The measurements were performed by applying a linearly varying voltage in the range of ±2 V to the top electrode with the bottom electrode grounded (Figure 1); current was changed within ± 4 mA. The applied voltage allowed creation of an electric field of the order of 1 MV/cm in the TiO_2_ layer. The Au circular-shaped electrodes were used as external point contacts. The contact area was about 1 ×10^−4^ cm^2^. The electrode holder allowed contact with a constant clamp force. Measurement was performed in a few steps (Figure 2): positioning of the electrode over measuring point, gentle landing of the electrode, carrying out desired sweeps/switching, lifting the electrode.

To study the atomic and electronic structure of the active layer of TiO_2_ at the point of impact of a high electric field (at the point of resistive switching) a special fiducial grid was applied on the surface of the sample, enabling measurement of the current-voltage characteristics and NEXAFS spectra in the same point of the film: the light spot on the sample was combined with contact area between the Au electrode and film TiO_2_. For this purpose the aluminum fiducial marks were deposited on the surface of pristine TiO_2_ by magnetron sputtering. All the marks were located on the same line.

The resistive switching in all the systems was performed outside the vacuum chamber of the spectrometer, in the air. Note that earlier [33] the possibility of switching in the air in all the studied samples was confirmed by implementing the resistive switching in a specially designed box under a nitrogen atmosphere. The resistive switching processes were shown to be almost identical in ambient and nitrogen atmosphere. A specially designed table with a vernier was used. The system allowed the position of the Au electrode to be controlled: it could be mechanically moved along a straight line. The resistive switching was carried out at the points located midway between the Al marks.

**Figure 3.**
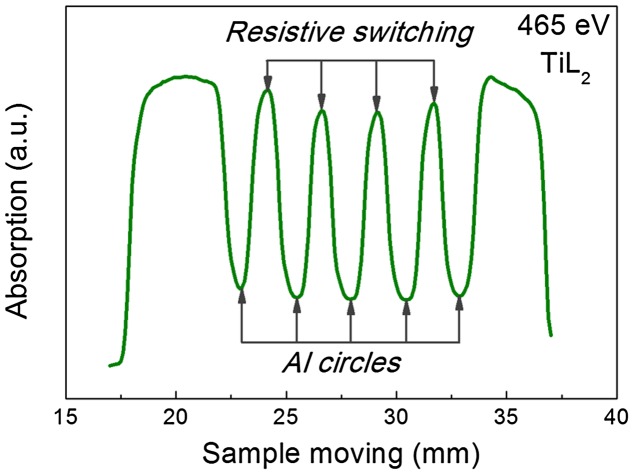
The example of scanning over the length of the sample carried out at 465 eV. The energy 465 eV corresponds to the Ti L_2,3_ absorption edge where a strong absorption by titanium atoms occurs. The minima on the curve correspond to the aluminum marks, maxima correspond to the TiO_2_ film located between aluminum marks.

After that the sample was placed in the vacuum chamber of the spectrometer so that a straight line passing through the centers of marks were coincided with the line at which the light spot could move during the translation the sample holder. Note that it was possible to move the sample perpendicularly to the beam during the experiment without upsetting the vacuum; this has permitted different points on its surface to be studied. In order to align the scale of the Au electrode movement in air with movement of the sample in the vacuum chamber, the scanning all over the length of the sample was conducted at fixed photon energy. We used an energy of 465 eV, which corresponds to Ti L_2,3_ absorption edge where a strong absorption by titanium atoms occurs. An example of such a scan is shown in Figure 3. The minima on the curve correspond to the aluminum marks, and the maxima correspond to the TiO_2_ film located between labels.

As a result the points at which the implementation of high electric field (resistive switching) was carried were established. Further, the Ti L_2,3_- and O K absorption spectra were measured at these points. Schematic representation of the idea of the experiment is shown in Figure 1.

The NEXAFS measurements were performed at the reflectometer set-up mounted on the optics beamline (D-08-1B2) of the Berlin Synchrotron Radiation facility BESSY-II of the Helmholtz Zentrum Berlin (HZB). A Hamamatsu GaAsP diode (4 x 4 mm window), together with a Keithley 617 electrometer, was used as a detector. NEXAFS spectra were measured at the incident angle of 45° in the vicinity of Ti L_2,3_ and O K absorption edges with energy resolution better than E/ΔE = 3000. The spectra were obtained by monitoring the total electron yield from the samples in a current mode. Apertures were used to narrow the area of the light spot on the film. Taking into account the size of X-ray beam which was 150 × 200 μm^2^ and the grazing incidence angle 45°, the size of the studied area was about 200 × 200 μm^2^. Thus the size of the studied area was approximately equal to the active area in which resistive switching occurs. It is important to emphasize that after applying the voltage the electrode was retracted from the film TiO_2_, and further NEXAFS measurements were carried out directly at the contact area at the TiO_2_ film without electrode. As a consequence, the obtained NEXAFS spectra provided information on changes in the electronic and atomic structure itself from the electric field impact area. The schematic representation of electrophysical and NEXAFS measurements is shown in Figure 1. The paper is focused on the Al/ITO/TiO_2_ assembly, which was studied before (as pristine) and after electric field effect. Additionally the deg-Si/ITO/TiO_2_ assembly was studied as a reference system.

## 3. Results and discussion

**Figure 4.**
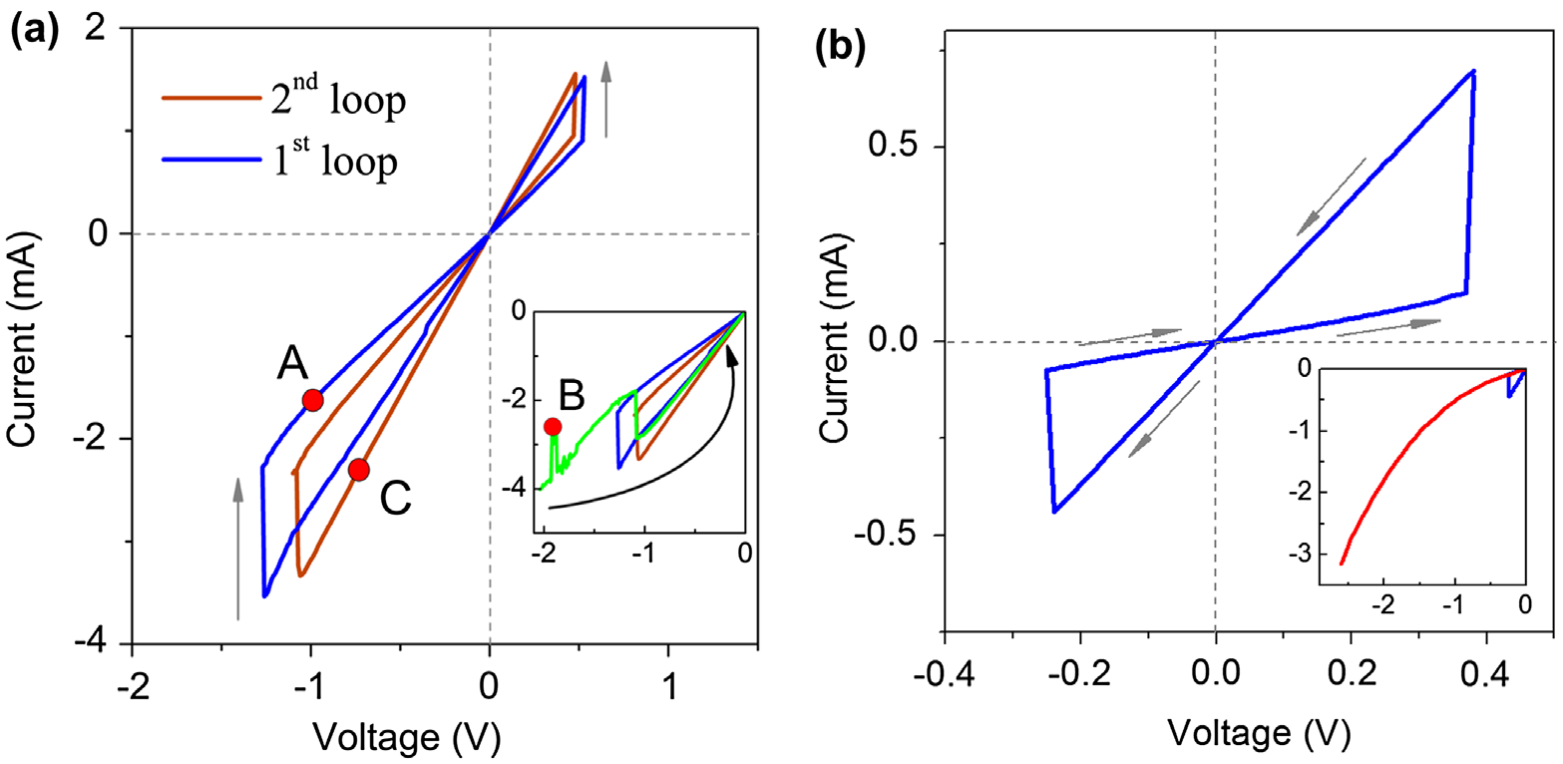
I-V characteristics for the substrate/ITO/TiO_2_ assemblies (a) Al substrate; (b) deg-Si substrate. In panel (a) the blue curve is a first stable hysteresis loop. The brown curve is a second stable hysteresis loop obtained right after the current instabilities in the green curve (inset). The inset zooms into the region where the current instability occurs. The green curve is one sweep from 0 to –2 V after obtaining the first stable hysteresis loop, current instabilities were observed starting from –1.7 V. Both hysteresis loops were repeatable at least 15 times with uncertainty of 0.5%. The red circles in the curves denote the steps of I-V sweep on which the NEXAFS measurements were performed. A, R_H_ state in the first loop; B, current drop in the region of instability; C, R_L_ state in the second loop. Inset in the panel (b) shows the sweep beyond the stable hysteresis loop, no instabilities were observed.

The stable reversible resistive switchings in the Al/ITO/TiO_2_ assembly were achieved by applying a linearly varying voltage. The stable hysteresis loop is shown in Figure 4(a) (blue curve). This state will be termed the first stable hysteresis loop. Only anti-clockwise bipolar resistive switching was realized. Since no instabilities were observed in the range of the stable hysteresis loop the sweep up to –2 V was performed beyond the voltage (–1.3 V) needed for the R_L_→R_H_ transition. The instabilities of current started from the –1.7 V. It is important that a current drop had occurred at –1.9 V. The voltage was switched off at –2 V after the reverse current jump. The current instability resulted in transition to a new hysteresis loop that is shown in the inset in Figure 4(a) (brown curve). An initial state in the new loop was R_L_. The values of R_L_ and R_H_ had decreased in comparison with the values for the initial hysteresis loop. It should be noted that the values of R_L_ and R_H_ established for first and second stable hysteresis loops were repeatable many times (at least 15 times) with an uncertainty of ±0.5%.

It was plausible to assume that the process of the current instability was accompanied by irreversible changes in electronic and atomic structure of the TiO_2_ because the initial hysteresis loop was no longer obtainable. In order to gain insight into the electronic and atomic structure of the active TiO_2_ layer the NEXAFS study was conducted at different steps of the electric field effect, which are shown in Figure 4(a) by red circles. Note that the 20 nm thick film was studied. Taking into account the value of a probing depth (of the order of 7 nm) [34,35] in the NEXAFS technique, it is obvious that we studied the processes occurring in the volume (closer to the surface) of the film but not at the interface between the TiO_2_ film and ITO. Actually we studied the response of the electronic and atomic structure of the film to the effect of electric field.

**Figure 5.**
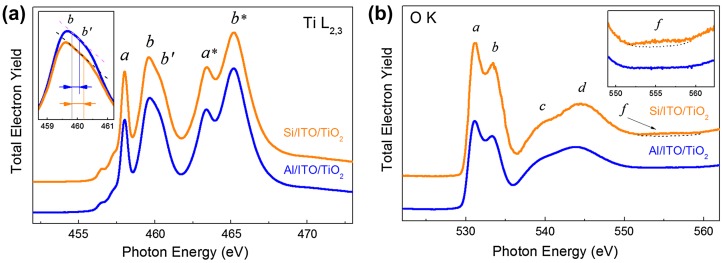
(a) Ti L_2,3_ (2p)- and (b) O K (1s)-absorption spectra of the Al/ITO/TiO_2_ and deg-Si/ITO/TiO_2_ assemblies (pristine structure). The insets in panels (a) and (b) are zooms of features ***b*-*b′*** and ***f*** respectively.

In the first step of the NEXAFS experiment the film (pristine structure) was studied at different points. The absorption spectra in the vicinity of Ti L_2,3_(2p) and O K(1s) absorption edges were measured (Figure 5(a) and (b)). It is important to emphasize that within the statistical error (in the order of 3%) the spectra measured at different points on the surface of the sample coincided.

According to the classical conception, the NEXAFS excitation at the Ti 2p threshold in TiO_2_ occurs mainly within the octahedral environment of oxygen atoms and the Ti 2p absorption spectrum should reflect the energies of the free Ti 3d states, because it is dominated by the 2p → 3d dipole transitions in the Ti atoms.[36] One can see that the Ti 2p absorption spectrum reflects clearly the spin-orbit splitting of the Ti 2p level. The Ti 2p_1/2_ structures are marked by asterisks in Figure 5(a). The peaks ***a*** and ***b*** in Ti 2p_3/2_ spectrum stem from dipole allowed transitions of Ti 2p_3/2_ electrons to unoccupied 3d states, which are split into 3dt_2 g_ (peak ***a***) and 3 de_g_ (peak ***b***) components by the octahedral crystal field created mainly by the O ions. The line shape analysis of the core-to-*e*_*g*_ transitions attracts the highest attention in order to reveal the crystal modification of TiO_2_ film. The absence of the splitting the peak ***b*** (doubly degenerated *e*_*g*_ component) indicates an amorphous state of the studied TiO_2_ film (Figure 5(a)). It should be noted that the Ti 2p- absorption spectra measured before and after the electric field effect were almost indistinguishable, which means the main changes of the structure occur in the sublattice of oxygen. Due to this reason only the O 1s-absorption spectra will be discussed further.

The relative intensities of all the O 1s-absorption spectra have been normalized to the continuum jump (at the photon energy of 560 eV) after subtraction of a sloping background, which was extrapolated from the linear region below O 1s-absorption onset. Such normalization provides about the same total oscillator strength for all the O 1s-absorption spectra over the photon energy range of 520–560 eV in accordance with a general idea of oscillator strength distribution for the atomic X-ray absorption.[36]

Figure 5(b) shows the O 1s-absorption spectrum of the pristine structure of the TiO_2_ film. According to [37–40], the molecular orbitals of TiO_2_ derived from a linear combination of atomic orbitals (LCAO) are characterized by four unoccupied orbitals: 2t_2 g_, 3e_g_, 3a_1 g_ and 4t_1u_. In TiO_2_, which has zero d-electrons, all four molecular orbitals are completely empty. In this classification the O 1s-edge features (labeled as ***a***, ***b***, ***c*** and ***d***) can be assigned to one electron transition from the O 1s orbital to the 2t_2 g_, 3e_g_, 3a_1 g_ and 4t_1u_ orbitals of TiO_2_, respectively. Thus ***a*** and ***b*** peaks reflect the core-electron transitions in the oxygen atoms to the lowest unoccupied Ti 3dt_2 g_ and 3 de_g_ electronic states that are mixed with the 2p states of the ligand (oxygen) atoms. The analysis of the energy position of the peaks shows that within the experimental accuracy (10 meV) the energy separation between ***a*** and ***b*** peaks is equal to 2.3 eV, which correlates well with the value for amorphous TiO_2_.[41,42] It is important to stress that according to [40] the feature ***c*** originates from the states, which are dominated by oxygen 2p character and they are best assigned as antibonding combinations of direct oxygen–oxygen interactions. The feature ***d*** can be assigned to titanium 4 sp band mixing with O 2p states. The absence of the feature at the 553 eV in the O 1s-absorption spectrum is additional evidence of the amorphous structure of the film.[42–44]

As it has been mentioned in the introduction, according to the model proposed in [28], the distribution of filaments in the switching matrix is generally random, which allows a large variation of switching voltages for different cells from time to time. One of the factors affecting the stability of the resistive switching is a microstructure of the active layer.[29–31] These authors found that with increase of crystallinity (decrease of grain boundary density), the resistances and switching voltages become much more stable. According to [32] the location and growth direction of the filaments are confined by the grain boundaries. Thus it is plausible to assume that amorphous structure of TiO_2_ film may be a reason for current instabilities observed.

**Figure 6.**
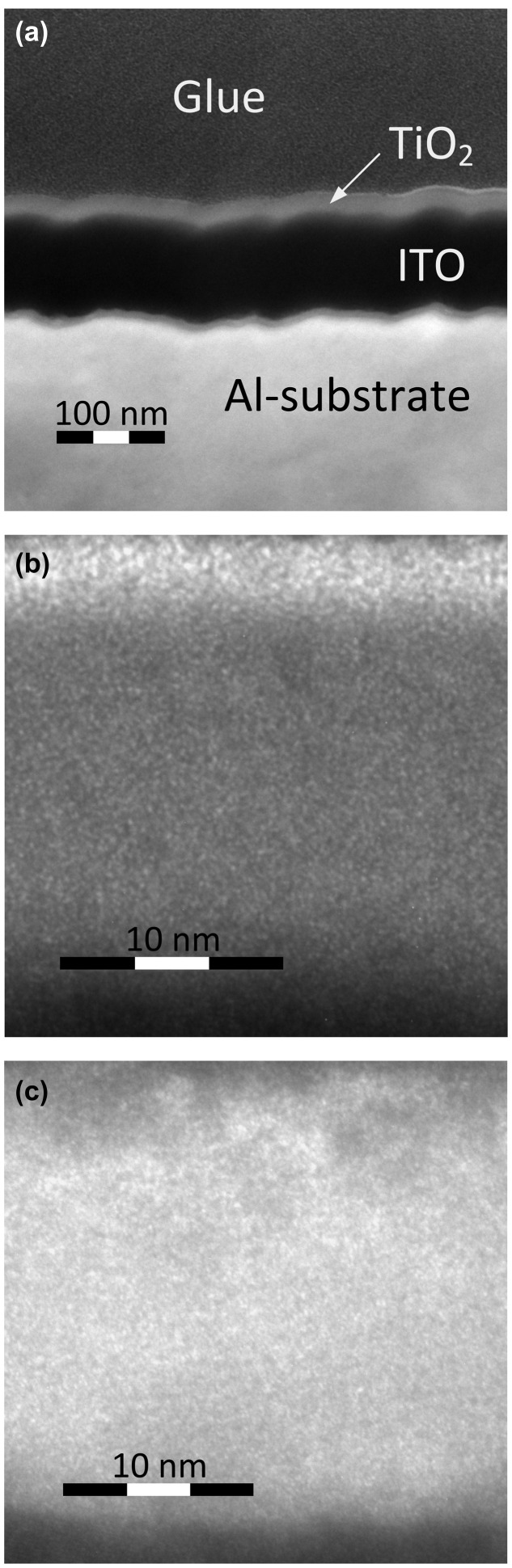
TEM image of Al/ITO/TiO_2_ assembly (a). Panels (b) and (c) show the HRTEM images of TiO_2_ film in Al/ITO/TiO_2_ and deg-Si/ITO/TiO_2_, respectively.

In order to gain further insight into a microstructure of TiO_2_ layer we have studied a surface topography of TiO_2_ film and Al substrate by helium ion microscopy (HIM) and high resolution transmission electron microscopy (HRTEM). The TEM image of Al/ITO/TiO_2_ assembly is shown in Figure 6(a). One can see that Al substrate has a pronounced surface relief with characteristic size no less than 20 nm. The ITO layer and TiO_2_ film replicate this relief. It is important that TiO_2_ film uniformly coats the surface without any discontinuity. The scaled HRTEM image in the area of TiO_2_ film is shown in Figure 6(b). No crystal order can be seen in the structure of the TiO_2_ film, which confirms its amorphous structure.

**Figure 7.**
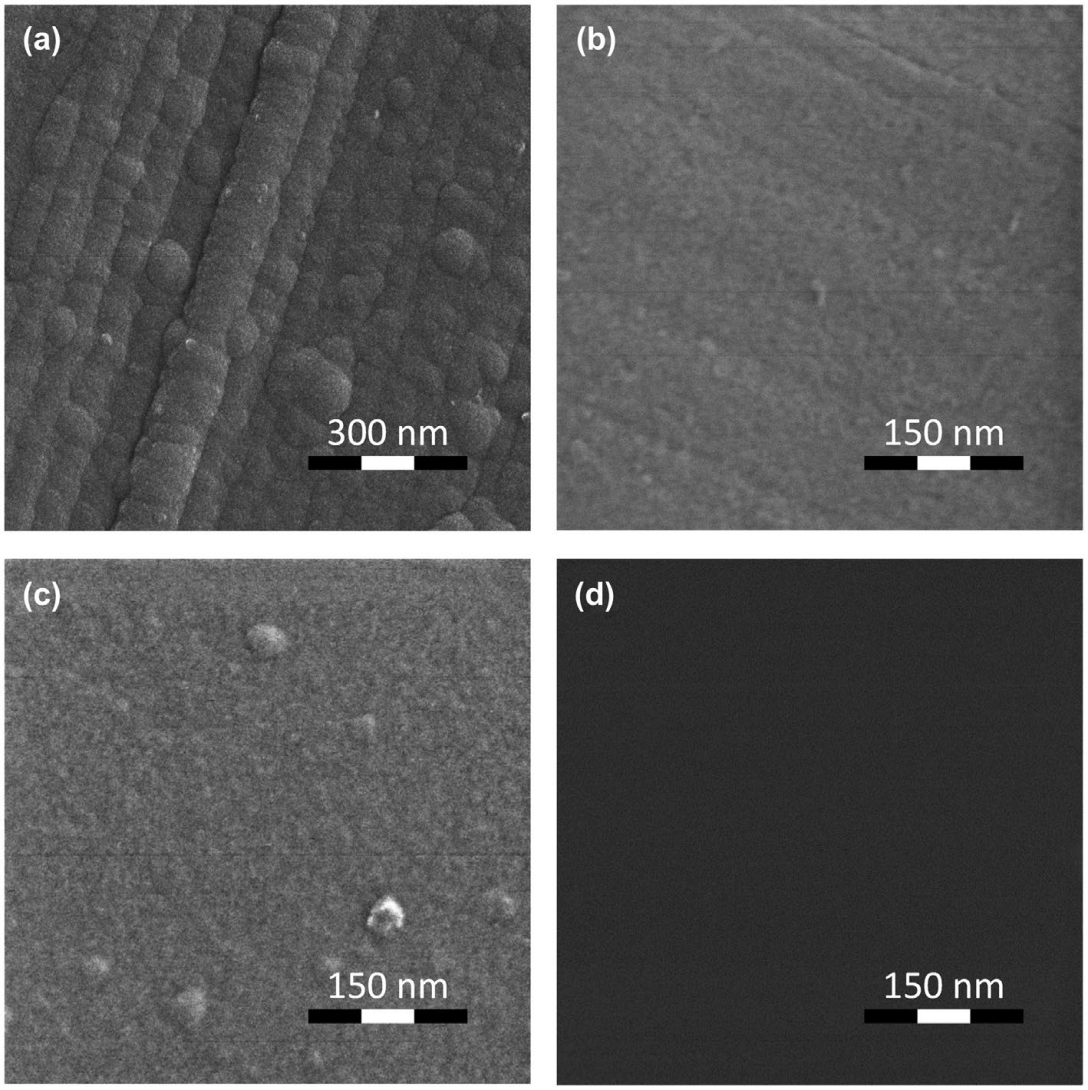
HIM image of TiO_2_ film deposited on (a) Al/ITO and (c) deg-Si/ITO substrates and of an Al substrate (b) and a deg-Si substrate (d).

The HIM image of the surface topography of the TiO_2_ film deposited on Al substrate is shown in Figure 7(a). The surface of the TiO_2_ film formed on the Al substrate reveals a grainy morphology with size of grains in the range 20–30 nm. It is important that a clearly defined relief of the surface in the form of rolled strips is traced. The depth of the groove is no less than 10–15 nm and occasionally reaches the value of 20 nm. The surface of the Al substrate has the same morphology features, particularly rolled strips (Figure 7(b)). One can presume that such substrate relief causes the grainy structure of the TiO_2_ film, leading to the current instabilities.

To support our idea that amorphous structure of the TiO_2_ film may be a reason for the observed current instabilities we have additionally studied the deg-Si/ITO/TiO_2_ assembly. The I-V characteristic for the deg-Si/ITO/TiO_2_ assembly is shown in Figure 4(b). No instability was observed in this system, either inside the hysteresis loop or beyond that, as is shown in the inset of Figure 4(b). As follows from Figure 7(c) the film grown on the deg-Si substrate shows a rather smooth uniform surface. Let us look at the Ti L_2,3_ absorption spectrum of this system in pristine state, shown in Figure 5(a). The spectrum of the film synthesized on deg-Si differs from the spectrum of film grown on the Al substrate in the contrast and the shape of the feature ***b*** (appearance of a shoulder ***b′***). This doubling of the Ti 2p→3 de_g_ transitions in TiO_2_ is well known [40,41,43–45] and is a result of symmetry breaking on Ti atom, which is typical for different crystal modifications (anatase, rutile, and brookite) of TiO_2_. The manifestation of doubling of the Ti 2p→3 de_g_ transitions depends on the crystalline structure of TiO_2_ and is expressed in the relative intensities of features ***b*** and ***b′***. In anatase the intensity of feature ***b*** is substantially higher than intensity of the feature ***b′***, while in rutile the opposite situation is observed.[43,45] Analysis of Ti L_2,3_ absorption spectra allows us to assume the presence of signs of an ordered structure of the anatase type in the film synthesized on deg-Si, as the shoulder ***b′*** is more pronounced for deg-Si/ITO/TiO_2_ (Figure 5(a) inset; the spectra shown here are normalized to the intensity of first peak ***a***). One can see a decreasing intensity of features ***b***-***b′*** relative to peak ***a*** for deg-Si/ITO/TiO_2_ assembly. According to [46,47], the intensity of *t*_*2*_ _*g*_ state (feature ***a***) dominates over *e*_*g*_ state (feature ***b*-*b′***) in the Ti L_2,3_ absorption spectra of crystalline TiO_2_, while the opposite situation is observed for amorphous TiO_2_. Thus the higher intensity ratio of *t*_*2*_ _*g*_ - *e*_*g*_ components (features ***a*** and ***b*-*b′***) may be considered as additional evidence of better order in TiO_2_ film in deg-Si/ITO/TiO_2_ assembly. The analysis of the shape of the O K absorption spectra of deg-Si/ITO/TiO_2_ and Al/ITO/TiO_2_ (Figure 5(b)) can further support our assumption. According to [46,47], the higher intensity of peaks ***a*** and ***b*** in Figure 5(b), together with their narrower width and shift of feature ***d*** toward the higher energy side in comparison with Al/ITO/TiO_2_, also points to a better structural order of TiO_2_ film in deg-Si/ITO/TiO_2_ assembly. Finally, the presence of the broad and weak feature ***f*** in the O K absorption spectrum of the TiO_2_ film grown on the deg-Si is an evidence of signs of ordered structure of the anatase type in this film.[40] In view of the electrophysical studies mentioned above, one can conclude the correlation exists between the crystallinity of the film structure and resistances and switching voltages stability that agrees well with [29–32].

Further, the film was studied after the electric field effect. The red circles in the I-V curves in Figure 4(a) denote the following steps of I-V sweep on which the NEXAFS measurements were performed: A denotes the R_H_ state in the first loop, B was chosen in the range of current instability, specifically after a current drop, and C was chosen after a reverse current jump, which had caused R_L_ state at new hysteresis loop.

**Figure 8.**
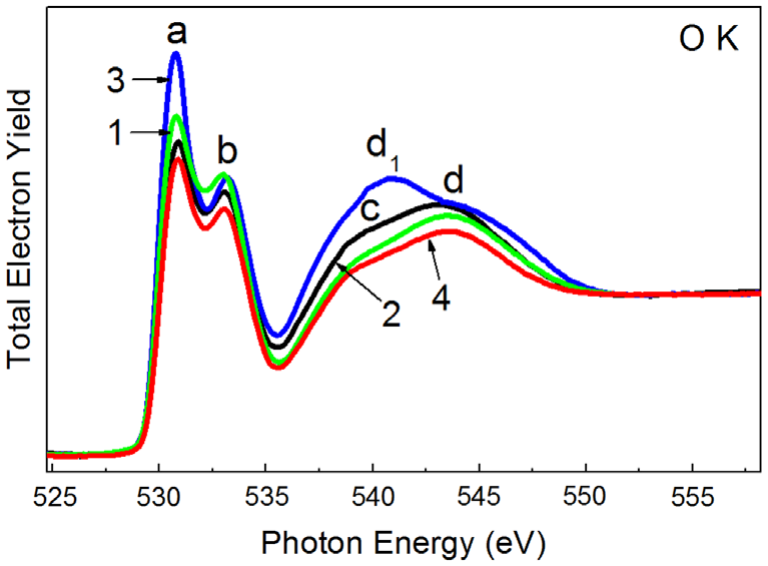
O K (1s)-absorption spectra of the Al/ITO/TiO_2_ assembly measured at different stages of the electric field effect, characterized by different values of current and voltage: 1, pristine structure; 2, R_H_ state at first hysteresis loop (after electroforming); 3, after current drop in the region of current instability; 4, R_L_ state at second hysteresis loop. The negative polarity of bias was used.

Figure 8 shows the O 1s-absorption spectra of the TiO_2_ film at the different steps of the electric field effect. In order to enable a comparison of the spectral features, the relative intensities of the spectra have been normalized to the same continuum jump at the photon energy of 560 eV, after subtraction of a sloping background, which was extrapolated from the linear region below the O 1s absorption onset. Such normalization provides about the same total oscillator strength for all the O 1s-absorption spectra over the photon energy range of 525–560 eV, in accordance with a general idea of oscillator strength distribution for the atomic X-ray absorption.[36] As it follows from Figure 8 the spectra correlate well in number and energy position with the main features of the fine structure. At the same time a clear change of the integrated intensity of peak ***a*** before and after inducing the electrical filed can be traced. Two different tendencies can be noticed. A significant intensity decrease is observed for peaks ***a*** and ***b*** depending on the step in orderly sequence: pristine structure, R_H_ state at first hysteresis loop (after electroforming) (A), and in the R_L_ state at second hysteresis loop (C). Contrary to these spectra the peak intensity ***a*** increases considerably and peak ***d***_***1***_ arises in the spectrum corresponding to the current drop (B) in the region of the current instability at the I-V characteristic.

Let us consider jointly the measured O 1s-absorption spectra and I-V characteristics. When a high electric field is applied to pristine structure (electroforming process) the oxygen atoms are knocked out from the lattice and move to the electrode with the positive voltage creating defects that causes formation of the conducting levels in the band gap. Note that in octahedral complexes the metal e_g_ orbitals (peak ***b***) are directed toward the oxygen atoms and have a strong overlap with the oxygen 2p orbitals. As a result, the O 1s-near edge structure is very sensitive to changes in the local environment of Ti atom.[43,48,49] The above-mentioned change of the integrated intensity of peak ***a*** before and after inducing the electrical field (decrease of the integrated intensity of peak ***a*** after electroforming process) together with the results of studies [48,49], according to which the peak ***a*** of the O 1s-absorption spectrum strongly depends on the concentration of oxygen vacancies, support the physical model of the electroforming process.

As follows from Figure 8 (4th curve), a subsequent decrease of the resistance (transition to R_L_ state at second hysteresis loop) is also accompanied by some decrease of the peaks intensity ***a*** and ***b***. It is plausible to assume that this transition is connected with further the oxygen atoms leaving the lattice and formation of the oxygen vacancies under the action of the electric field. The electric field stimulates the directed oxygen ions drift from the region of the formed vacancy localization, providing a spatial separation of the vacancy and oxygen ion preventing the possibility of reverse capture. The shift of the oxygen ions O^2–^ can occur also due to diffusion that can be promoted by the local heating.

**Figure 9.**
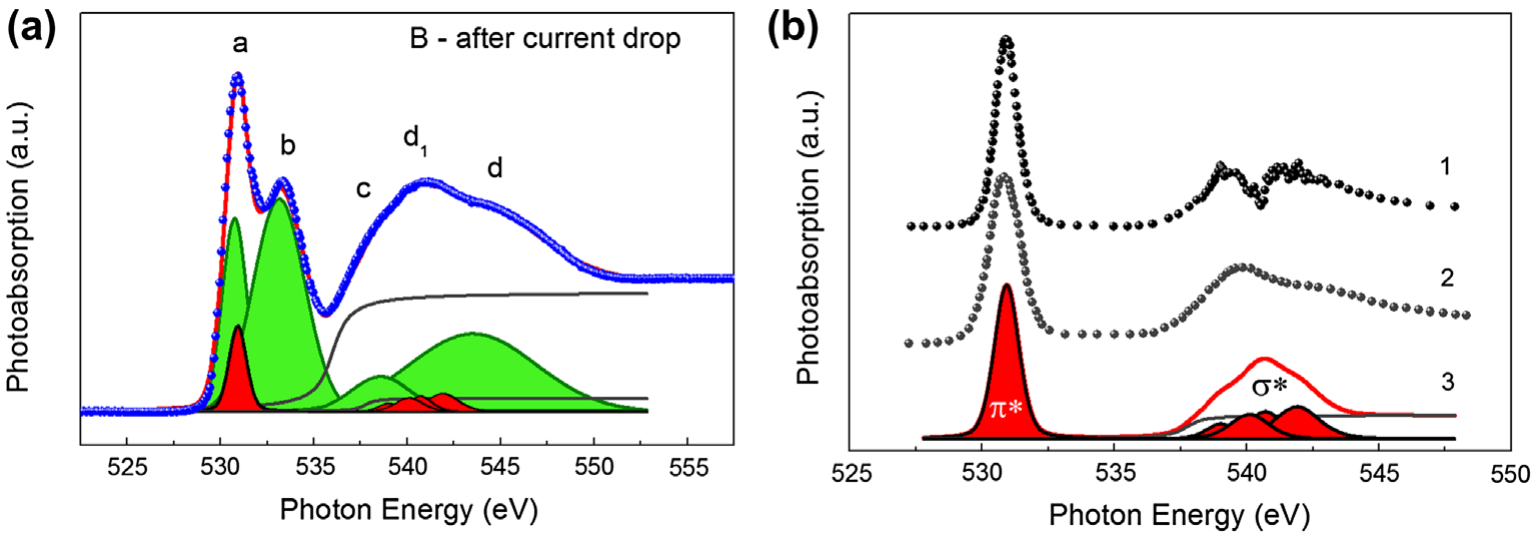
(a) O 1s-absorption spectrum of the Al/ITO/TiO_2_ assembly measured in the region B after current drop in the region of the current instability. The deconvolution of the spectrum was performed using the free software “Athena” [50]. Green peaks correspond to t_2 g_, e_g_, a_1 g_, and t_1u_ states of TiO_2_; red peaks correspond to π* and σ* states of molecular oxygen. (b) 1 and 2, O 1s-absorption spectra of molecular oxygen in gas-phase and physisorbed on Pt(111) respectively from [51]; 3, a resulting curve for sum of π* and σ* peaks of molecular oxygen in deconvolution of O 1s-absorption spectrum shown in (a).

Figure 9(a) shows the O 1s-absorption spectrum of the TiO_2_ film measured after the current drop in the region of the instability at the I-V characteristic. The deconvolution of the spectrum performed using free software “Athena” [50] is also shown. The symmetrical Gaussian shape of peaks was used. The continuous edge jump at ionization potential has been represented by the arctangent function. The position of edge jump 536 eV was estimated from sum of O1s binding energy [52] and work function [53] in TiO_2_. It can be seen from the presented deconvolution that the discussed spectrum is a sum of two spectra. The first one correlates with the spectrum of amorphous TiO_2_.[41,42] The second spectrum demonstrates the presence of a localized narrow peak and four rather wide peaks. The first narrow peak correlates well in width and energy position with the first sharp resonance in absorption spectra of molecular O_2_ presented in [51,55] which is due to1σ_u_→1π_g_* transition. The spectra of molecular O_2_ contain the second relatively broad doublet band which is related to the 1σ_g_→3σ_u_* excitation. Doubling of this band results from the spin-up and spin-down configurations in the final state. The doubling of this band and its relative intensity depend on the state of O_2_ but it is essential that the energy position of the center of the σ* band is not changed for gas-phase O_2_, condensed or physisorbed O_2_ on the metal surface. A resulting curve for sum of π* and σ* peaks in deconvolution of spectrum measured in the region of current instability (Figure 9(a)) is shown in Figure 9(b) in comparison with the spectra of gas-phase O_2_ and O_2_ physisorbed on Pt(111) from [51]. It is important that the center of σ* band in the resulting envelope curve is almost the same as that in the presented spectra of gas-phase O_2_ and physisorbed O_2_. This allows us to affirm that molecular O_2_ is being formed in the TiO_2_ bulk during current instabilities. The absence of actual doubling of the σ* band in the resulting envelope curve may indicate that not exactly gaseous O_2_ is being formed in TiO_2_ at the moment of current instability but some intermediate form of O_2_. The bond length is almost the same for the intermediate and gaseous O_2_ forms and these intermediate O_2_ molecules are not chemically bonded to TiO_2_ matrix, as the energy separation between π* and σ* bands and the shape of σ* band are significantly different for lengthened O_2_ molecules as it takes place in case of chemisorbed oxygen.[51] A formation of titanium dioxide molecule dimers (TiO_2_)_2_ seems also not to be the case. It is shown in [56] that titanium dioxide molecule dimers have symmetry significantly lower than TiO_6_ octahedra. Changing the symmetry of Ti surroundings would surely provoke changing the shape of Ti L_2,3_ absorption spectra but actually the shape of those was almost unchanged at all studied stages of electric field effect, as mentioned above.

The formation of O_2_ molecules was indirectly indicated in a number of studies [57–60] by means of observation of bubbles on the surface of the top electrode of some assemblies after resistive switching. All knowledge of the occurrence of oxygen gas has till now been obtained from scanning electron microscopy (SEM) or atomic force microscopy (AFM) images and does not give a clear idea at which specific stage of the switching process the formation of molecular oxygen occurs. One can see from our result that the O_2_ molecules are formed exactly in the region of instability of current because neither the O 1s-absorption spectrum after electroforming nor that for R_L_ state in the second loop contains the contribution of O_2_ molecules (Figure 8). One can see that the influence of electric field on the film in this region leads to formation of the molecular oxygen that is accompanied by the momentary decrease of the current (Figure 4(a)).

**Figure 10.**
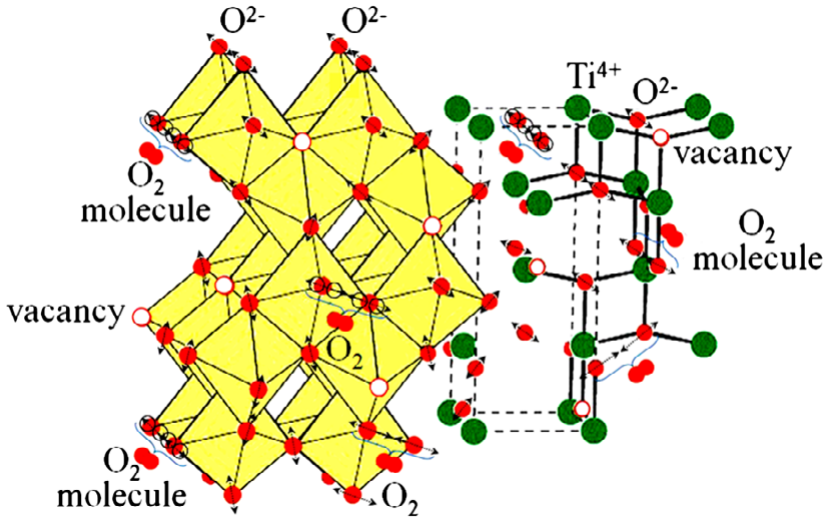
Schematic presentation of the oxygen atoms leaving the lattice, formation of the oxygen vacancies under the action of electric field and local heating and formation of the O_2_ molecules in the Al/ITO/TiO_2_ assembly.

In our opinion the observed phenomenon is a manifestation of the joint action of electric field and local heating (self-heating), where the dominant mechanism is heating, wrenching the oxygen atoms off the regular bonds with formation of the oxygen vacancies. In reality the further increase of the voltage up to the region of instability leads to the increase of the amplitude of the oxygen atoms vibrations due to electrical Joule heating and as a result to the creation of the O_2_ molecules. We have established that the observed current instability does not depend on the direction of the electric field, which confirms our supposition. The instability occurs at a voltage specific to the system. Exactly at these voltages the creation of the O_2_ molecules occurs. Figure 10 summarizes the described processes and gives a schematic presentation of the formation of the O_2_ molecules in the Al/ITO/TiO_2_ assembly.

## 4. Conclusions

We have characterized an Al/ITO/TiO_2_ assembly, where a TiO_2_ layer of 20 nm thickness was grown by ALD. The electric field effect on the TiO_2_ structure was studied by NEXAFS in addition to electrophysical measurements. We have observed current instability in the I-V characteristics and have shown its independence on the direction of the electric field. It has been revealed that this current instability is supported by the amorphous structure of the TiO_2_ film but is initiated by the surface morphology of the Al substrate. A formation of the O_2_ molecules in the TiO_2_ active layer has been established specifically in the region of current instability. The formation of the oxygen bubbles on the surface of the top electrode of some assemblies during resistive switching was reported in [57–60]. All the knowledge of occurrence of oxygen gas has until now been obtained from SEM or AFM images and does not give a clear idea at which specific stage of the switching process the formation of molecular oxygen occurs. It has been only noted that the occurrence of the bubbles impedes the stable reversible resistance switching.

In the present studies we have directly observed formation of the O_2_ molecules using NEXAFS spectroscopy and have shown that formation of the O_2_ molecules occurs specifically in the region of the current instability as a result of electrical Joule heating manifestation. The obtained results are very important for a deeper understanding of the mechanism of resistance switching and charge transport in TiO_2_ films and its future application as switching material in resistance change memory based devices.

## Disclosure statement

No potential conflict of interest was reported by the authors.

## Funding

This work was supported by the Helmholtz-Zentrum Berlin
